# Cannabinoid receptor CB1 regulates STAT3 activity and its expression dictates the responsiveness to SR141716 treatment in human glioma patients' cells

**DOI:** 10.18632/oncotarget.3895

**Published:** 2015-05-11

**Authors:** Elena Ciaglia, Giovanni Torelli, Simona Pisanti, Paola Picardi, Alba D'Alessandro, Chiara Laezza, Anna Maria Malfitano, Donatella Fiore, Antonio Christian Pagano Zottola, Maria Chiara Proto, Giuseppe Catapano, Patrizia Gazzerro, Maurizio Bifulco

**Affiliations:** ^1^ Department of Medicine and Surgery, University of Salerno, Baronissi Salerno, Italy; ^2^ Department of Pharmacy, University of Salerno, Fisciano, Salerno, Italy; ^3^ ”G.Rummo” Medical Hospital, Department of Neurosurgery, Benevento, Italy; ^4^ Neurosurgery Unit A.O. San Giovanni di Dio e Ruggi d' Aragona - Salerno's School of Medicine, Largo Città di Ippocrate, Salerno, Italy; ^5^ Institute of Endocrinology and Experimental Oncology, IEOS CNR, Naples, Italy; ^6^ Department of Biology and Cellular and Molecular Pathology, University of Naples Federico II, Naples, Italy

**Keywords:** STAT3, CB1, MICA, NK cells, gliomas

## Abstract

Herein we show that a majority of human brain tumor samples and cell lines over-expressed cannabinoid receptor CB1 as compared to normal human astrocytes (NHA), while uniformly expressed low levels of CB2. This finding prompted us to investigate the therapeutic exploitation of CB1 inactivation by SR141716 treatment, with regard to its direct and indirect cell-mediated effects against gliomas. Functional studies, using U251MG glioma cells and primary tumor cell lines derived from glioma patients expressing different levels of CB1, highlighted SR141716 efficacy in inducing apoptosis via G1 phase stasis and block of TGF-β1 secretion through a mechanism that involves STAT3 inhibition. According to the multivariate role of STAT3 in the immune escape too, interestingly SR141716 lead also to the functional and selective expression of MICA/B on the surface of responsive malignant glioma cells, but not on NHA. This makes SR141716 treated-glioma cells potent targets for allogeneic NK cell-mediated recognition through a NKG2D restricted mechanism, thus priming them for NK cell antitumor reactivity. These results indicate that CB1 and STAT3 participate in a new oncogenic network in the complex biology of glioma and their expression levels in patients dictate the efficacy of the CB1 antagonist SR141716 in multimodal glioma destruction.

**SIGNIFICANCE:**

CB1 is implicated in the regulation of cellular processes linked to survival, proliferation, invasion and angiogenesis in several physio-pathological conditions. We shed light on previously unrecognized molecular mechanism of CB1-mediated modulation of human glioma progression and provide the first and original demonstration of CB1-STAT3 axis as a new target and predictor biomarkers of the benefit from specific therapies. Indeed CB1 antagonism capable of tumoral cell division' control while making the glioma immunovisible and engaging the immune system to fight it may represent a hopeful alternative to other established chemotherapeutics. Because different aspects of glioma biology have been separately targeted with very limited success, we speculate that CB1 inhibitors which enclose in the same molecule cytotoxic potential and high activity to boost competent immune surveillance mechanisms, at a degree that seems to be correlated to the levels of CB1 immunoreactivity, might have profound implications for exploring new therapeutic anti-glioma actions.

## INTRODUCTION

Glioblastoma multiforme (GBM) is a fatal disease characterized by uncontrolled cellular proliferation, diffuse infiltration, a tendency for necrosis, significant angiogenesis, intense resistance to apoptosis, and widespread genomic aberrations. Despite years of research in anti-tumoral therapeutic strategies, malignant glioma remains one of the most aggressive forms of cancer, with a median survival after resection, radiotherapy and chemotherapy of 12–15 months [1]. To improve the treatment outcome of this devastating disease, new therapies are then urgently needed.

The ubiquitous regulatory action of the endocannabinoid system in health and disease emphasizes the role of this endogenous system in several physio-pathological processes and makes its pharmacological manipulation a promising strategy for the management of many different diseases, including cancer. Indeed, this system, composed of cannabinoid receptors CB1 and CB2, their endogenous ligands (endocannabinoids, eg, anandamide) and the enzymes for their metabolism, has been shown to be involved in the control of proliferation, migration, and invasive behavior of a wide variety of tumor types [2]. In glioma the engagement of CB receptors by THC and other cannabinoid agonists has been reported to inhibit tumor growth in animal model through a well established mechanism of action that seems to be active in patients, too [3]. However little is currently known about the biological role of the endocannabinoid system in cancer physiopathology and many basic questions remain unanswered.

In the central nervous system the main effects of the endocannabinoid system rely on the activation of CB1, a 7-transmembrane-domain protein belonging to the G_αi_ protein-coupled receptor family (GPCR) which to date has been shown to be involved in memory and learning processes, in diseases affecting movements, mood and anxiety and in conditions related to altered brain reward mechanisms [4]. It is also expressed and functional active in neuronal progenitors, where it regulates cell proliferation and differentiation [5–6]. Some studies showed that the endocannabinoid tone and CB1 receptor expression are higher in tumor tissues than in non malignant tissues; additionally this trend has been associated with disease severity and outcome, suggesting that an altered endocannabinoid tone may be pro-tumorigenic. In support of this, CB1 receptor immunoreactivity has been correlated with a more severe form of the disease at diagnosis and poorer outcome in prostate cancer [7] and pancreatic ductal adenocarcinoma patients [8]; Messalli et al. have recently proved that its expression increased from benign and borderline to malignant epithelial ovarian tumors [9] and further, a high CB1 receptor immunoreactivity is indicative of a poorer prognosis in stage II colorectal cancer patients [10]. As regard to glioma, discrepancies in CB1 receptor expression were observed by different groups. Held-Feindt et al. reported no significant change in mRNA expression of CB1 receptor in glial tumors versus normal tissues [11], whilst more recently Wu et al. attested an increase of CB1 receptor expression in high-grade glioma, compared with low-grade glioma and non-tumor brain tissues [12]. In the light of this background and as CB1 receptor was recently suggested as a potential oncogene in pediatric sarcoma [13], its function in glioma progression deserves much attention. Indeed the evidence that the endogenous ligand anandamide is founded upregulated in glioblastoma brain tissue [14] suggests that the endocannabinoid system may be over-activated in cancer, leading us to investigate the therapeutic exploitation of CB1 receptor inactivation as a promising anti-glioma strategy. In particular, SR141716 is a potent and selective CB1 receptor antagonist. Interestingly, besides its antagonist properties SR141716 can exert also inverse-agonist effects exhibiting a significant anti-tumor action in thyroid, mantle cell lymphoma, fibrosarcoma, leukaemia, breast and colon tumors both *in vitro* and *in vivo* [15–20] while its complete functional significance in glioma has remained not fully explored, especially for its immunomodulatory effects.

The highly lethal nature of glioblastoma suggests that the levels of immunogenic signals by glioma cells are to low to induce an antitumor immunity. Then, among potential novel therapies, combined chemoimmunotherapy remains an attractive approach for GBM patients. Recent studies have shown that GBM may be vulnerable to elements of the innate immune system through its expression of several MHC class I-like stress-associated molecules, such as MHC class I chain-related proteins A and B (MICA/B) and human cytomegalovirus membrane glycoprotein (UL-16)-binding proteins [21]. These antigens are recognized by Natural Killer (NK) cells via the stimulatory receptor NK group 2 member D (NKG2D) using innate mechanisms that are MHC-independent and do not require prior antigen exposure or priming [22]. Thus, the immunity to glioma may be boosted by achieving high levels of activating NKG2D ligand on the surface of cancer target cells. In the last few years, increasing evidence have indicated that efficient chemotherapeutic agents can induce specific immune responses that result in immunogenic cancer cell death or immunostimulatory side effects [23]. In this study we found an upregulation of CB1 in human glioma tissues and primary cell lines which correlates with the activity status of STAT3. Moreover, the inactivation of this oncogenic axis directly affects human glioblastoma and also stimulates NK cell-mediated antitumor effects. Indeed, according to the role of STAT3 in the promotion of survival and proliferation, but also in the immune escape of cancer cells, SR141716, besides a direct antiproliferative potential, specifically induces expression of NKG2D ligand MICA/B in malignant but not in healthy neuronal cells, leading to a specific stimulation of NK-antitumor immune response at a degree that seems to be correlated to the levels of CB1 immunoreactivity.

## RESULTS

### The pharmacological inactivation of CB1 receptor by SR141716 induces apoptosis through G1 phase block in human glioma cell lines *in vitro*

**Figure 1:**
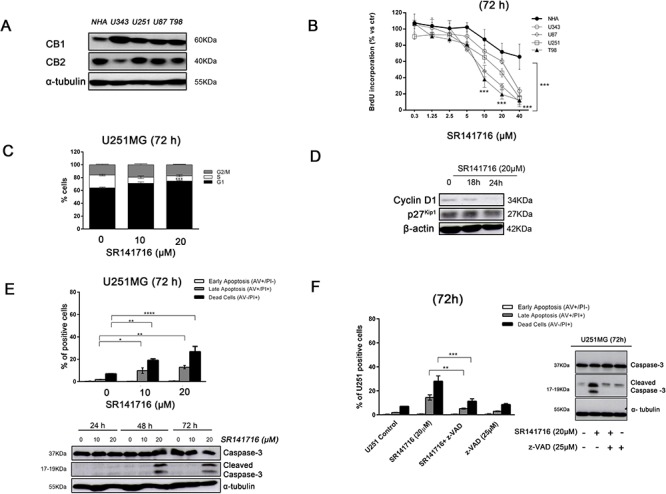
Effect of SR141716 on growth and cellular integrity of human glioma cell lines and primary astrocytes **A.** Basal expression of CB1 and CB2 and in normal human astrocytes (NHA) and different glioma cell lines (U343, U251, U87 and T98). Panel shows a representative western blot of 3 different experiments performed with similar results. α-tubulin serves as loading control. **B.** Glioma cell lines and NHA were cultured for 72 h in the presence of the indicated concentrations (0–40 μM) of SR141716 before analysis of cell proliferation by BrdU incorporation assay. Results are expressed as means ± SD of 3 independent experiments performed in triplicate and reported as percentage *vs* the untreated control (ANOVA, ***P < 0.001 *vs* control). **C.** Distribution of U251 glioma cells in the different phases of the cell cycle in SR141716-treated (10–20 μM) cells and in parallel untreated cultures. Histograms show the percentage of cells in each phase of the cell cycle. Results are representative of 3 independent experiments performed in duplicate, expressed as mean ± SD (ANOVA, ***P < 0.001 *vs* control). **D.** Time-dependent expression of cell cycle regulators cyclin D1 and p27^Kip1^ detected by Western blot. U251cells were treated with SR141716 (20 μM) for 18 h and 24 h. β-actin serves as loading control. Data are representative of 3 independent experiments performed with similar results. **E.** Induction of apoptosis measured by annexin V and propidium iodide (PI) double staining through flow cytometry in SR141716-treated U251 cells. Histograms indicate total percentage of early (Annexin V-positive cells/PI-negative cells) and late apoptotic events (Annexin V/PI-double positive cells) as well as necrotic cells (Annexin V-negative cells/PI-positive cells). Results are representative of 3 independent experiments performed in duplicate and expressed as mean ± SD (ANOVA, *P < 0.05, **P < 0.01 and ***P < 0.001) (*upper panel*). Concentration- and time-dependent increase of the apoptotic pathway by expression of cleaved caspase-3 determined by Western blot analysis. U251 glioma cells were treated with SR141716 (10–20 μM) for 24, 48, and 72 h. α-tubulin serves as loading control. Panel shows a representative blot of 3 different experiments performed with similar results *(lower panel)*. **F.** Apoptosis induction *(left panel)* and caspase 3 cleavage (*right panel*) measured, as in *E*, in U251MG cells pretreated for 2 h with vehicle (DMSO, control) or the indicated concentrations of z-VAD-fmk and then treated with SR141716 (20 μM) for 72 h.

We examined the protein levels of CB1 and CB2 receptors in 4 different glioma cell lines (U343, U251, U87, T98) and in normal human astrocytes (NHA) as their normal counterpart. The results showed that protein expression of CB1 receptor was up-regulated in all glioma cell lines tested compared with normal astrocytes. Conversely, CB2 immunoreactivity varied without a specific trend between normal and malignant cells (Fig. 1A). The altered expression of CB1 in glioma cells allowed them to be selectively targeted by CB1 receptor antagonist SR141716, whose pharmacological and biological effects we sought to explore. First, all glioma cell lines and NHA were incubated with increasing concentrations of SR141716 (0.3–40 μM) in time course. Specifically after 72 h-treatment, SR141716-exposed glioma cells showed a dose-dependent inhibition of proliferation compared with untreated cells (Fig. 1B). In particular, a clear reduction of proliferative rate became more apparent at a concentration of 20 μM, as demonstrated by the BrdU incorporation assay. Of note, SR141716 elicited no significant reduction of NHA proliferation as compared to cancer cells in the same condition. To gain better insight into the biological processes modulated by SR141716 treatment in glioma cells, U251 cell line was used as a model system for further studies, as this cell line is one of the most aggressive glioblastoma cell lines with a considerable expression of CB1. SR141716 was used in the subsequent experiments at the efficacious concentrations of 10 and 20 μM. At first, we investigated cell cycle distribution following SR141716 treatment for 72 h. As shown in Fig. 1C, the percentage of cells in G1 phase was significantly higher in U251 glioma cells treated with SR141716 (10–20 μM) than in untreated cells. Furthermore, a marked reduction of the cell population in the S phase was observed after SR141716 treatment, reaching its significance only at the highest dose of 20 μM. However, no significant differences in G2/M population were found. These results suggested that the antiproliferative effects of SR141716 in U251 cells can be related to a G1/S transition inhibition. Indeed, in SR141716-treated cells the protein levels of G1/S-specific Cyclin D1 were selectively reduced compared to control, with no detectable effect on the cyclin-dependent kinase inhibitor p27^kip1^ (Fig. 1D). To assess whether the inhibition of cell proliferation by SR141716 was also associated to the induction of apoptosis, we performed a cytofluorimetric cell death analysis by Annexin-V and propidium iodide staining. We observed a slight increase in apoptosis induction in glioma cells treated with SR141716 20 μM (Fig. 1E, *upper panel*). Accordingly, in control glioma cells and U251 treated with the lowest dose of SR141716 (10 μM), we didn't detect processed caspase-3. On the contrary, in response to SR141716 20 μM, caspase 3 was cleaved into the intermediated p20 form and its active p17 subunit after 48 h of treatment and sustained until 72 h (Fig. 1E*, lower panel*). As expected the broad spectrum caspase inhibitor z-VAD-fmk completely inhibited apoptosis induction (Fig. 1F, *left panel*) and caspase activation (Fig. 1F, *right panel*) in response to 72 h treatment with SR141716 20 μM. Taken together, the results illustrate that SR141716 affects glioma cell growth through the inhibition of cell-cycle progression and the induction of caspase-dependent apoptosis at the highest concentrations.

### Exposure to SR141716 renders U251 glioma cells more susceptible to NK cell-mediated cytotoxicity through the upregulation of the NKG2D ligand MICA/B

These observations, along with previously published data by our group [15–19], raise the concern that a potential antitumor effect might be ascribed to SR141716 in the context of glioma. Increasing evidence indicates that cancer cell perturbation induced by chemotherapeutic agents promote antitumor immune responses and contribute to their full clinical efficacy. To characterize if SR141716 was able to regulate immune surveillance mechanisms, we analyzed the effect of SR141716 treatment on the expression of NK- cell-activating ligands on U251 glioma cells and in turns on NK cell recognition and cytotoxicity against these cancer cells as target.

**Figure 2:**
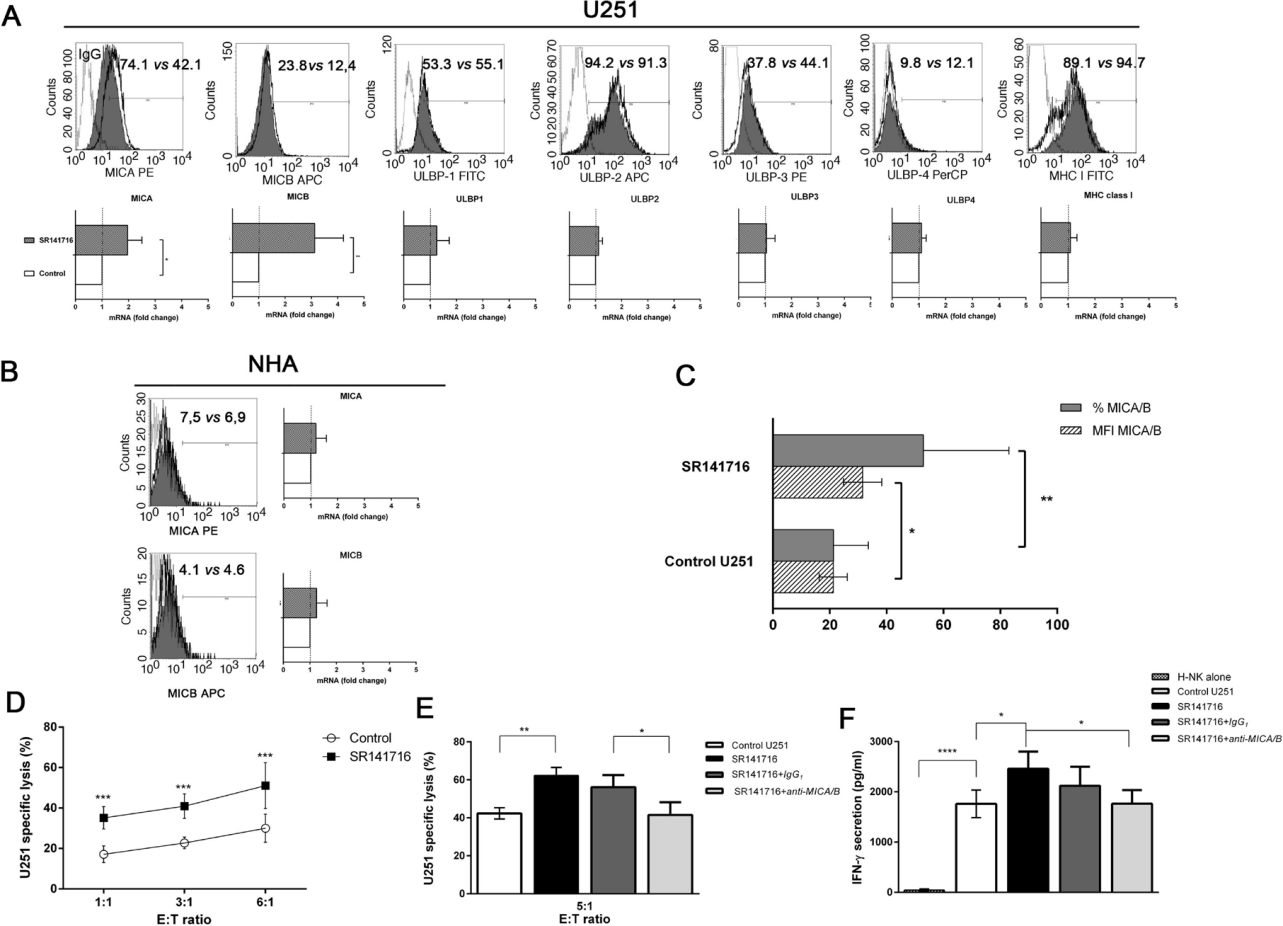
Phenotypic and functional significance of the upregulated expression of MICA/B on glioma cells treated with SR141716 **A.** Representative example for cytofluorimetric histogram profiles of MHCI, MICA, MICB, ULBP1, ULBP2, ULBP3, ULBP4 protein levels at the U251 cell surface of control (gray profiles) or cells treated with SR141716 20 μM for 24 h (empty profiles). Percentages of positive cells are indicated in the upper right corner and are representative of 3 independent experiments (*upper panel*). Real-time PCR analysis of total mRNA obtained from U251 cells, unstimulated or treated with SR141716 (20 μM) for 6 h. MHCI, MICA, MICB, ULBP1, ULBP2, ULBP3, ULBP4 mRNA expression were assessed. Data, expressed as fold change units, were normalized with GAPDH and referred to the untreated cells considered as calibrator. The mean of 4 independent experiments is shown (*P < 0.05, **P < 0.01 two-tailed Student's *t* test) (*lower panel*). **B.** Representative example for cytofluorimetric histogram profiles of MICA and MICB (*left*) and real-time PCR analysis (*right*) of the total mRNA obtained from NHA cells, unstimulated or treated with SR141716 (20 μM) as indicated in *A*. Data, expressed as fold change units, were normalized with GAPDH and referred to the untreated cells considered as calibrator. The mean of 4 experiments is shown. **C.** The mean fluorescence intensity (MFI) and the percentage of U251 positive cells for MICA/B were calculated based on at least 6 independent experiments and evaluated by ANOVA (*P < 0.05, **P < 0.01 compared with untreated cells). Bar graphs report mean values ± SD. **D-F.** SR141716 enhances NK cell-mediated cytotoxicity against U251 glioma cells. U251 cells were incubated for 24 h with SR141716 (20 μM) or control medium (untreated) prior to 4 h flow cytometric assay of NK-cell cytotoxicity at the indicated effector to target ratios (E:T), as described in Materials and Methods **E** and **F** Afterwards, supernatants were harvested and analyzed for IFN-γ by ELISA **F.** To evaluate the role of MICA/B, where indicated, blocking anti-MICA/B (SR141716+*anti-MICA/B*) or isotype control F(ab')_2_ fragments (SR141716+*IgG_1_*) were added before addition of NK cells **E** and **F**. Results were expressed as the mean ± SD of 4 independent experiments conducted in triplicate. All pairwise comparisons are statistically significant (ANOVA; *P < 0.05, **P < 0.01, ***P < 0.001).

First, we monitored by flow cytometry the cell surface expression of the NKG2D ligand MICA, MICB, ULBP1, ULBP2, ULBP3, ULBP4 and MHC class I on untreated and SR141716-treated U251 glioma cells at 24 h to exclude that the possibility of observed effects are linked to the stress of dying cells. FACS analysis revealed that U251 cells expressed the different antigens at various levels, showing high level of MHC class I antigen expression. We found that the incubation with SR141716 induced a substantial and selective up-regulation of MICA and MICB surface expression on U251 cells (Fig. 2A, *upper histograms*). Importantly, MHC class I surface expression was not altered by SR141716 treatment demonstrating that the observed changes were not due to an unspecific effect of the compound. The observed increase in MICA/B expression was both in terms of percentage of positive cells and Mean Fluorescence Intensity (MFI) (Fig. 2C), leading us to verify that a possible mechanism underlying MICA/B upregulation on U251 cells could be the consequence of an increased mRNA expression. Indeed, we found a significant increase of either MICA and MICB mRNA levels in treated cells with, as expected, no significant effect on the other NKG2D ligand mRNA levels (Fig. 2A, *lower panel*). It is of importance that, in contrast to the effects on cancer cells, MICA and MICB, in terms both of protein surface and mRNA levels, on NHA were not affected by SR141716 treatment (Fig. 2B), suggesting that SR141716 was able to specifically upregulate “danger signals” for NK cells in malignant but not in healthy cells. Then, it was still important to verify the functionality of MICA/B and further investigate if the SR141716-dependent increase in MICA/B expression could enhance NK cell recognition and killing of U251 glioma cells. As expected, treatment of U251 cells with SR141716 significantly increased specific killing (Fig. 2D) and production of IFN-γ by co-cultured NK cells (Fig. 2F), when compared with the cytotoxicity and cytokine secretion of control untreated cells. These increases were significantly dependent on MICA/B-NKG2D interaction, because considerably reduced in the presence of a blocking anti-MICA/B mAb, whereas isotype control had no significant effects (Fig. 2E–2F). Of note, MICA/B-blocking mAbs didn't affect basal cell lysis, indicating that constitutive NK cell degranulation and U251 glioma cell lysis probably does not involve NKG2D activating receptor.

These preliminary results therefore indicate that SR141716 might have additional therapeutical immunomodulation properties related to the specific increase of MICA/B expression.

### SR141716 treatment determines regression of glioma *in vivo*

To evaluate SR141716 efficacy also *in vivo*, we tested SR141716 effect on glioma tumor growth in a subcutaneous xenograft model. The U251 cells did not show a considerable tumorigenicity in nude mice in the initial attempts to establish xenograft model. Thus, we used U87 cells because this cell line has been widely used due to their higher tumorigenicity reported in nude mice.

**Figure 3:**
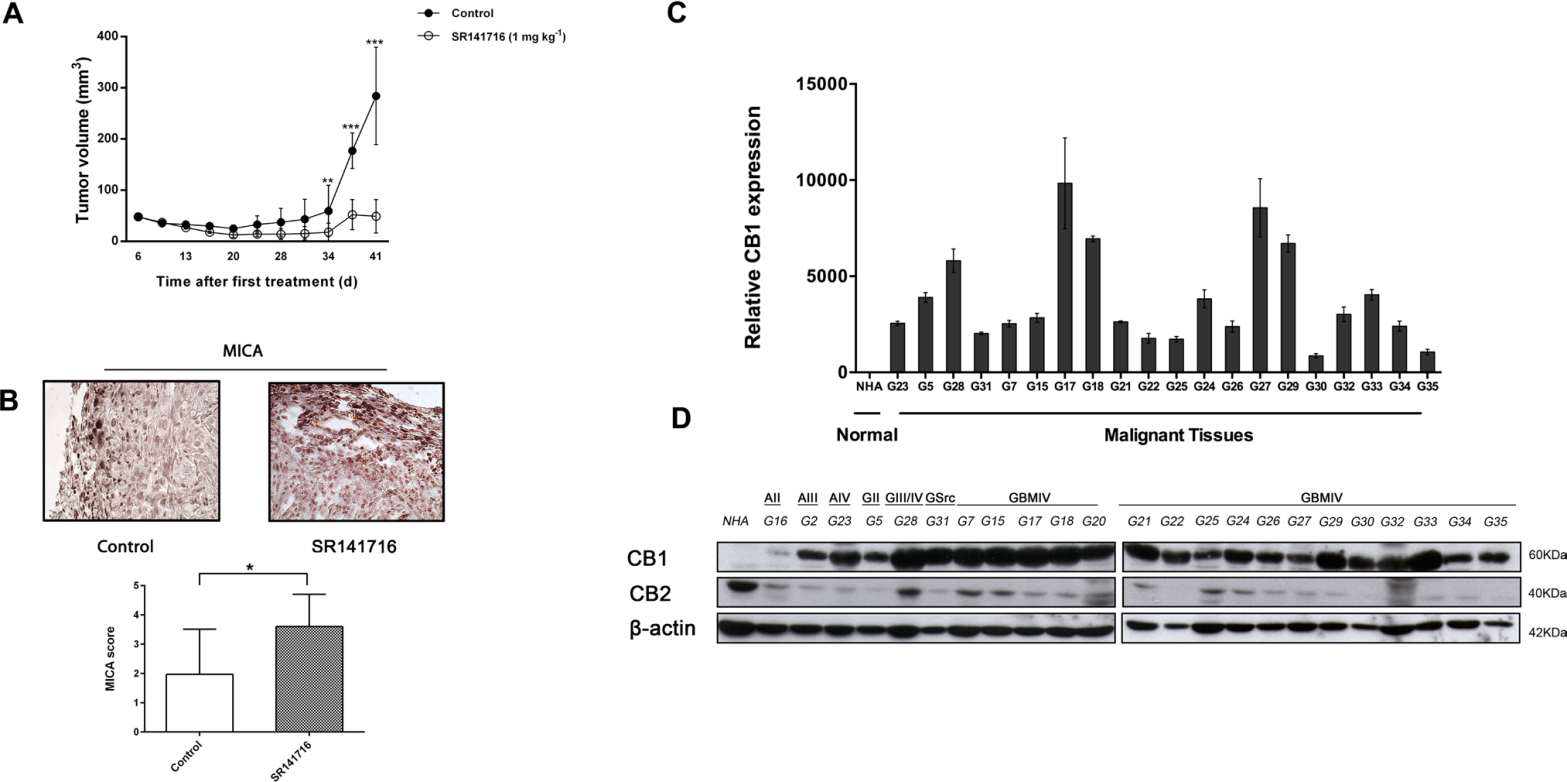
Analysis of CB1 expression and of the effects of its pharmacological modulation in an *in vivo* setting *SR141716 reduces the growth of glioma xenograft*. **A.** Effect of SR141716 20 μM on the growth of U87 cell-derived tumor xenografts [*n* = 10–12 for each condition; mean ± SD; SR141716-treated tumors were significantly different from vehicle, from day 34 until the end of the treatment (**P < 0.01, ***P < 0.001)]. **B.** Criosections (0, 7 mm) of luc-U87 explanted masses prepared at day 35 from vehicle (control) and SR141716-treated animals were stained with mAb directed against MICA. Magnification for all section was 20x and are representative of at least 3 different tumor sections for each treatment (*upper panel*). After 35 and 41 days from treatment beginning, 4 animals for each group were sacrificed for immunohistological analysis. Bar graphs in lower panel summarize IHC scores of MICA in xenograft specimens (*P < 0.05, two-tailed Student's *t* test) as reported in *Material and Methods*. *CB1 is upregulated in gliomas tissues and primary cell lines compared with NHA*. **C.** Real-time PCR analysis of CB1 in NHA and 20 glioma tissues (G5-G35). Data, expressed as fold change units, were normalized with β-actin and referred to the NHA considered as calibrator. Columns represent mean ± SD of the results performed in triplicates. **D.** Representative Western blot showing the basal protein levels of CB1 and CB2 in NHA and 23 tumor brains (G16 astrocytoma grade II, G2 astrocytoma grade III, G23 astrocytoma grade IV, G5 glioma grade II, G28 glioma grade III, G31 gliosarcoma, G7-G35 glioblastoma grade IV). β-actin was used as loading control.

U87 cell suspension was injected s.c. into nude mice and when the tumor size reached approximately 4–6 mm in diameter, 12 mice in SR141716 group received each the peritumoral injection of SR141716, at 1mg/Kg/dose, while 12 mice in control group received vehicle alone (PBS). We recorded the tumor sizes on the first day of SR141716 treatment (day 1) and biweekly at the indicated time points. The peritumoral injections of the drug were then repeated two times a week for 4 weeks. As results, all of the mice in vehicle (control) group developed tumors beyond 200 mm^3^ on average by day 37. In contrast, the mice in SR141716 group developed much smaller tumors (Fig. 3A). Specifically on day 34, 37, 41, the mice in the SR141716 group had significantly smaller tumor sizes compared with the mice in the control group. Furthermore, after 35 and 41 days from treatment beginning, 4 animals for each group were sacrificed for immunohistological analysis that revealed that SR141716 upregulated the expression of MICA in the tumor xenograft compared to the control group (Fig. 3B). Bar graphs in lower panel (Fig. 3B) summarize scoring results from the IHC analyses.

### CB1 is highly expressed in brain tumor samples and patients' primary glioma cells

**Figure 4:**
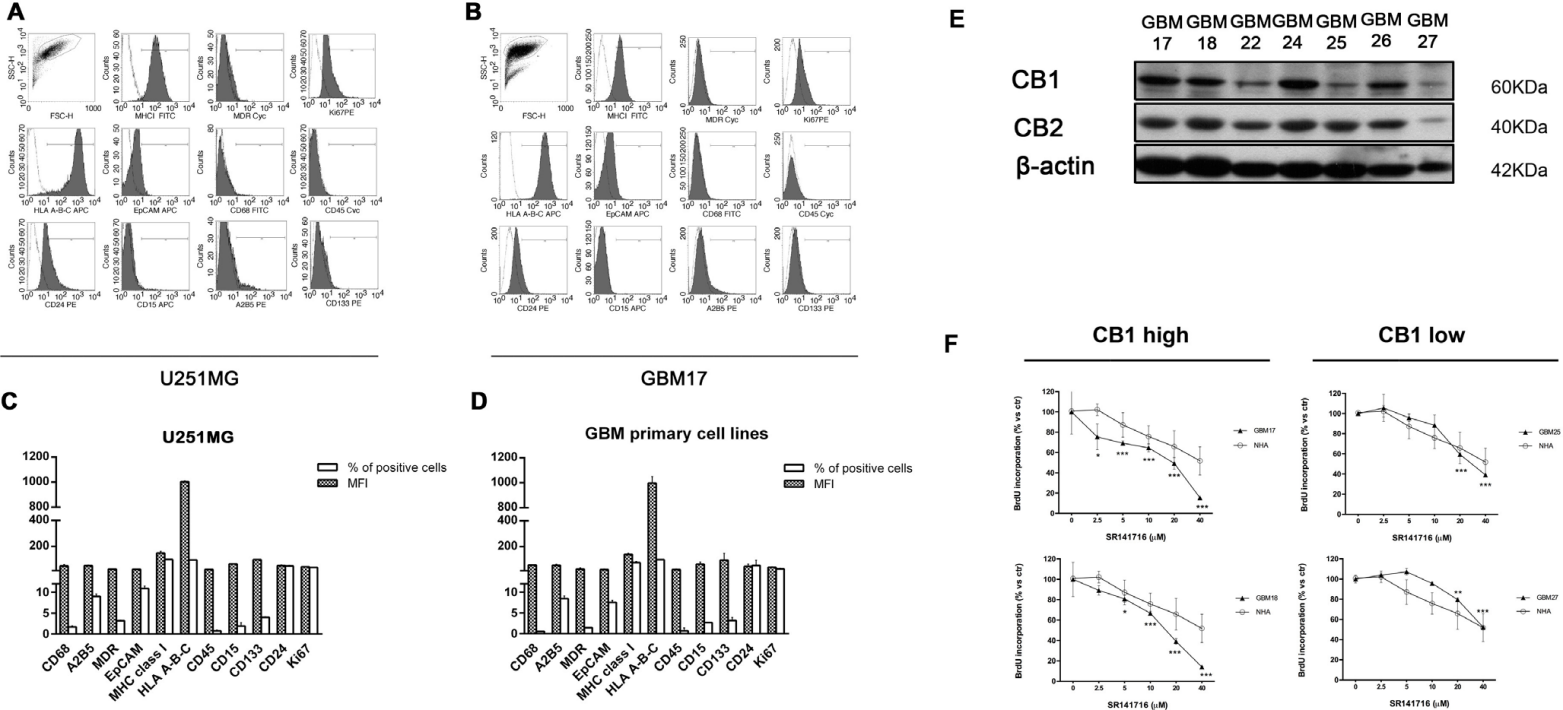
CB1 expression in glioma patients derived-cells dictates the responsiveness to SR1417176 treatment Phenotypic characterization of glioma primary cell lines. **A-D.** Glioma (GBM) primary cell lines used throughout the paper and U251for comparison were stained with the indicated antibodies followed by flow cytometric analysis. In all the experiments the isotype-matched controls were used to set up the negative values. **A-B.** Representative example for cytofluorimetric histogram profiles of U251 *A* and GBM17 **B** maintained in culture for 48 h (gray profiles). Open dot histograms represent isotype matched immunoglobulin staining. **C-D.** Bars graph reports mean ± SD of the mean fluorescence intensity (MFI) and percent of positivity for each marker in U251 **C** and in all different GBM primary cell lines used **D**. Results are representative of 3 independent experiments. **E.** Representative Western blot showing CB1 and CB2 protein levels in 7 human primary glioma cell lines established from the indicated cancer patients (G17, G18, G22, G24, G25, G26, G27). β-actin was used as loading control. **F.** Patients-derived primary cell lines were divided in two different groups reflecting their CB1 protein expression level [(GMB17, GBM18, GBM24, GBM26 CB1 *high*), (GBM22, GBM25, GBM27 CB1 *low*)]. 2 representative primary cell lines belonging to CB1 *low* (GBM25 and GBM27) and CB1 *high* (GBM17 and GBM18) cell groups respectively, and NHA were cultured for 72 h in the presence of the indicated concentrations (2.5–40 μM) of SR141716 20 μM before analysis of cell proliferation by BrdU incorporation assay. Results, reported as percentage, are expressed as mean ± SD of 3 independent experiments performed in triplicate. (ANOVA, **P < 0.01, ***P < 0.001 *vs* control).

As a new potential chemotherapeutical agent with combined anti-glioma action we then sought to verify the clinical relevance of SR141716 in terms of real sensitivity to its growth inhibiting and immunomodulatory effects. Since CB1 receptor is the most relevant pharmacological target of SR141716, we looked for a possible correlation between receptor expression levels in patients and drug activity. First, we examined the expression pattern of CB1 in gliomas, by extracting total RNA and proteins from 23 primary glioma samples, among which there were grade II, grade III, and grade IV glioma tissues, compared with normal human astrocytes. To avoid confounding effects of therapy, only samples from treatment-naïve patients, that is without prior radiation therapy and chemotherapy, were considered (Supplementary Table 1). Real-time PCR and Western blot analysis were then performed to evaluate gene expression and protein profiles. The results showed that mRNA and protein expression of CB1 was upregulated in glioma tissue compared with NHA (Fig. 3C–D). A strong positive signal of CB1 was found in almost all glioma tissues, whereas an almost undetectable level of CB1 in NHA was observed. As control, CB2 receptor levels did not show any apparent differences between normal and malignant tissues and/or association with tumor grade and were lower than CB2 expression in NHA cells (Fig. 3D). Then we addressed the functional relevance of this molecular finding, verifying if the results achieved for SR14176-treated U251 cells were also true for the primary cell lines established from fresh resectioned tumors of selected individual glioma patients. Preliminary FACS analysis suggested a similar overlapping phenotypic profile of all patients' derived primary cells used and U251 for comparison, corroborating that they had the features of human glioma cells (Fig. 4A-4D). After verifying the expression of CB1 and CB2 in the 7 established primary cell lines (Fig. 4E), we then moved to select individual glioma patient-derived cells with different CB1 receptor protein levels to characterize their sensitivity to SR141716 treatment on the basis of these receptors levels. Interestingly the primary cell lines derived from high receptor levels tumors (GBM17, GBM18) were more susceptible to the growth inhibiting effects of SR141716 than those derived from low to moderate receptor levels tumors (GBM25, GBM27) (Fig. 4F). These preliminary observations support that CB1 strongly positive cells are more likely to respond to SR141716 treatment *in vitro*.

### High amounts of CB1 predict the sensitivity of primary glioma patient-derived cells to SR141716 treatment

**Figure 5:**
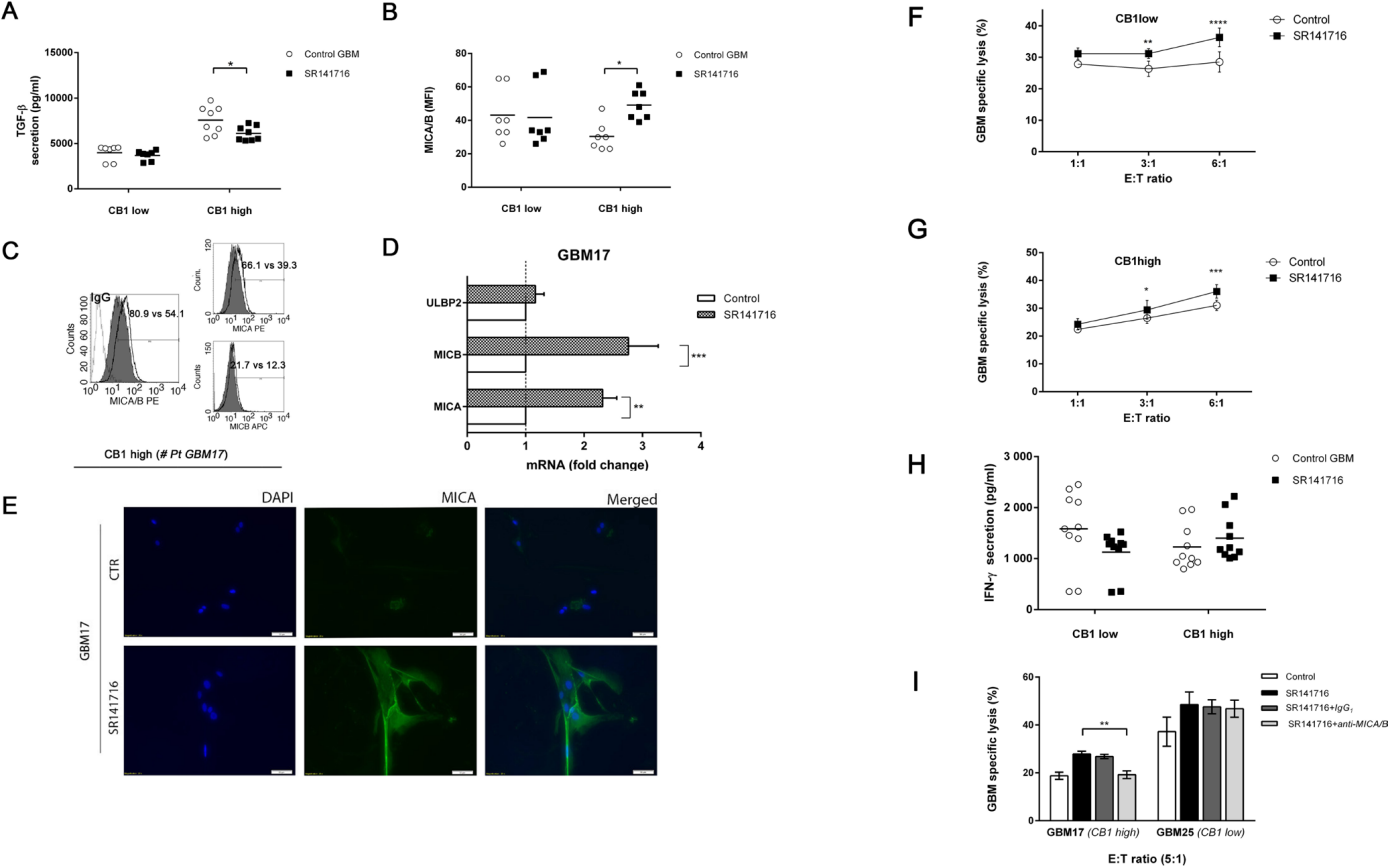
SR141716 stimulates MICA and MICB transcription and cell surface expression on responsive CB1 high primary cell lines enhancing NK-cell mediated cytotoxicity against glioma patient cells **A.** CB1 *low* (GBM22, GBM25, GBM27 CB1 *low*) and CB1 *high* (GMB17, GBM18, GBM24, GBM26) cell tumor primary cell lines were cultured for 24 h in the presence or absence of SR141716 20 μM before the determination of total TGF-β in cell culture supernatant by ELISA. Each dot represents the result from at least 2 different independent experiments for each indicated patient primary cell lines. **B.** MICA/B cell surface expression was analyzed by flow cytometry on CB1 *low* and CB1 *high* cells treated with SR141716 (20 μM) for 24 h. The mean fluorescence intensity (MFI) of MICA/B was calculated based on at least 2 different independent experiments for patient and evaluated by ANOVA (*P < 0.05, compared with untreated cells, *control GBM*). Each dot represents the result from at least 2 different independent experiments for each indicated patient primary cell lines. Bar graphs report mean values ± SD. **C.** Cell surface expression of total MICA/B or alternatively of MICA and MICB was analyzed by flow cytometry on one representative responsive patient's primary cell line (# GBM17) treated with SR141716 20 μM for 24 h. The gray-colored histograms represent basal expression of tot MICA/B, MICA or MICB whereas thick black-colored histograms represent the antigen expression after treatment with SR141716. **D.** Real-time PCR analysis of total mRNA obtained from GBM17 cells, unstimulated or treated with SR141716 20 μM for 6 h. MICA, MICB and ULBP2 mRNA expression was assessed. Data, expressed as fold change units, were normalized with GADPH and referred to the untreated cells considered as calibrator and represent the mean of 4 experiments (ANOVA, **P < 0.01, ***P < 0.001, compared with untreated cells, *control*). **E.** A representative patient's primary cell line (# GBM17) was cultured for 24 h in the presence or absence of SR141716 (20 μM). Subsequently, immunofluorescence analysis using the LEAF™ purified anti-human MICA/MICB (6D4) specific mAb followed by the secondary Alexa Fluor^®^ 488-coniugate and DAPI for nuclear staining was performed. One representative experiment of a total of 3 is shown. Magnification, 20x. **F-I.** CB1 *low F* and CB1 *high G* cell groups were incubated for 18 h with or without SR141716 20 μM prior to 4 h flow cytometric assay of NK-cell cytotoxicity at the indicated effector to target ratios (E:T), as described in Materials and Methods. Afterwards, supernatants were harvested and analyzed for IFN-γ by ELISA. Each dot represents the result from at least 2 different independent experiments for each indicated patient primary cell lines **H**. To evaluate the role of MICA/B, as indicated in *D*, blocking anti-MICA/B (SR141716+*anti-MICA/B*) or isotype control F(ab')_2_ fragments (SR141716+*IgG_1_*) were added before addition of NK cells **I.** Results were expressed as the mean ± SD of 4 independent experiments conducted in triplicate. All pairwise comparisons are statistically significant (ANOVA; *P < 0.05, **P < 0.01, ***P < 0.001).

Next we verified if the results achieved for SR14176-treated U251 cells were also true for the patient derived primary cell lines and significantly differ between high- and low-CB1-expressing cells. First, given that anti-proliferative activity of SR141716, we hypothesized that SR141716 might also regulate transforming growth factor-β1 (TGF–β1) production, a cytokine upregulated during gliomas malignant progression. In particular TGF-β1 isoform may contribute to tumor pathogenesis by direct support of tumor growth, self-renewal of glioma initiating stem cells and stimulating expression of the vascular endothelial growth factor as well as the plasminogen activator inhibitor and some metalloproteinases that are involved in vascular remodeling, angiogenesis and degradation of the extracellular matrix [24–25]. All these promoted the investigation of SR141716 effects on TGF-β1 levels in our expermental model. Really we noticed a decreased level of TGF–β1 secretion in supernatant of high-CB1-expressing cells after treatment with SR141716 but not in that of low-CB1-expressing primary cell lines in the same conditions (Fig. 5A). TGF–β1 also interferes with several mechanisms of anti-tumor immune responses and selectively down-regulates MICA expression [26]. Accordingly the incubation with SR141716 induced a substantial and selective up-regulation of MICA/B surface expression in high- but not in low-CB1 expressing primary cell lines (Fig. 5B). In particular, as reported for one representative patient primary cell line, belonging to the responsive high-CB1 group (GBM17), SR141716 treatment mainly upregulates the basal expression of MICA, at both mRNA (Fig. 5D) and surface protein level (Fig. 5C) with less pronounced but equally significant effects also on MICB (Fig. 5C–5D). At the immunofluorescence analysis, a weak MICA expression was detected on untreated glioma GBM17 cells while, of course, it was much higher after 24 h treatment with SR141716 (Fig. 5E). Surprisingly, we did not observe any significant differences in the degree of specific killing and production of IFN-γ by NK cells in the coculture with high- and low-CB1-expressing cells treated with SR141716. In fact, a slight difference in the degree of IFN-γ production was observed between the two different groups even though it failed to reach statistical significance (Fig. 5H) and of note, accordingly SR14176 treatment increased susceptibility to NK cell killing of both high (Fig. 5F) and low receptor levels cell lines (Fig. 5G) when compared with the cytotoxicity of control untreated cells. However only for high-CB1-expressing cell lines the increase in NK cell lysis was significantly dependent on MICA/B-NKG2D interaction; indeed in GBM17 patient primary cell line (high-CB1-expressing) but not in GBM25 patient primary cell line (low-CB1-expressing) a considerable NK specific lysis reduction in the presence of a blocking anti-MICA/B mAb was achieved (Fig. 5I). These data suggest that the augmented expression of MICA in high-CB1-expressing cell lines treated with SR141716 enhances NK cell activation and killing but we cannot rule out the effect of SR141716 on other molecules or ligands that can modulate NK cell recognition for the control of low grade malignancy.

### Pathway activation signature of SR141716 effects in human glioma: a role for STAT3

**Figure 6:**
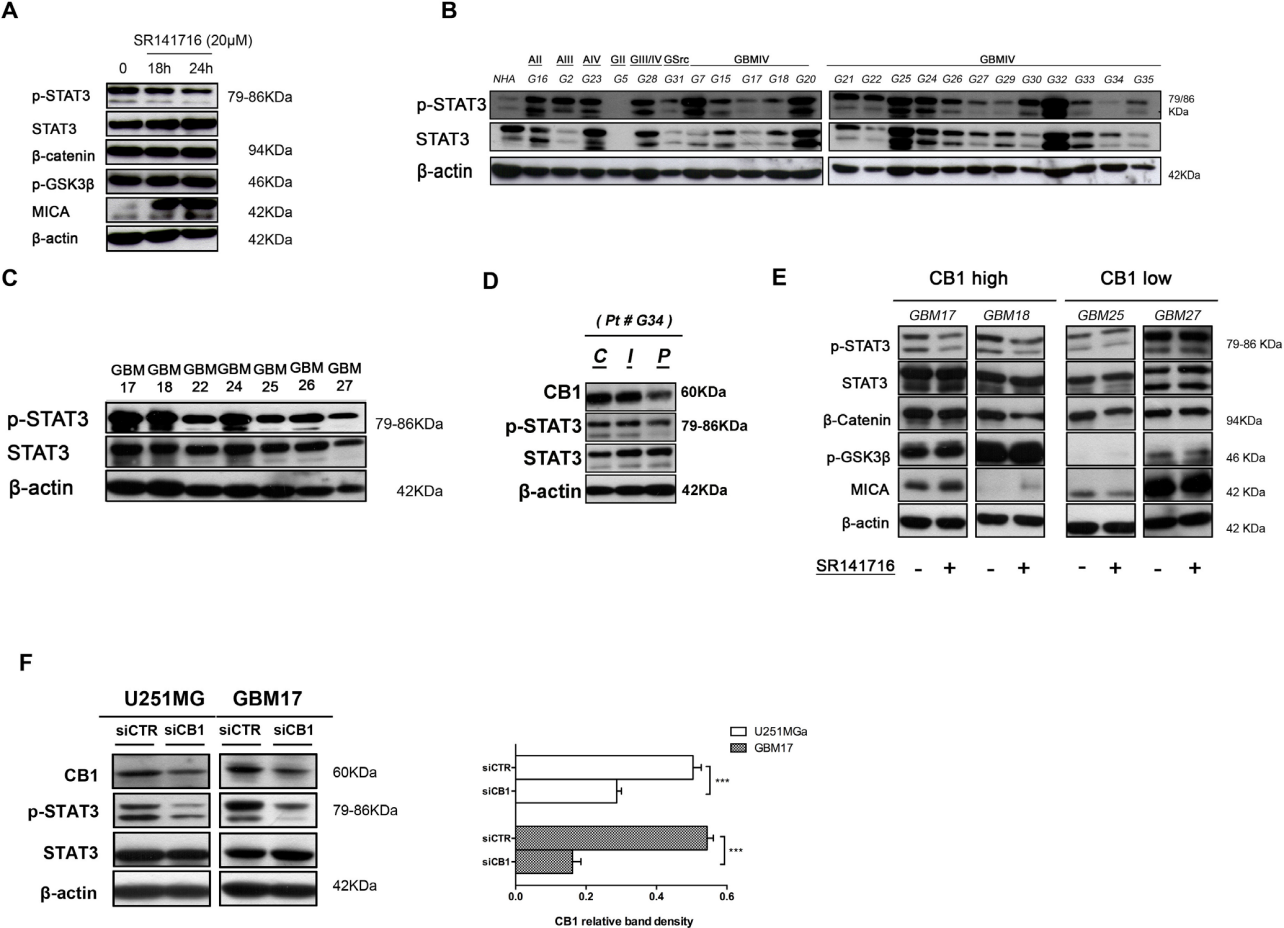
Cannabinoid receptor CB1 specifically regulates STAT3 activity **A.** Inhibition of STAT3 pathway by CB1 targeting. U251 cells were stimulated for the indicated time with SR141716 20 μM and cell lysates were immunoblotted for p-STAT3, total STAT3, β-catenin, p-GSK3β, MICA and β-actin as loading control. Data are representative of 3 independent experiments performed with similar results. **B.** Representative Western blot showing the basal protein levels of p-STAT3 and total STAT3 in NHA and 23 tumor brains (G16 astrocytoma grade II, G2 astrocytoma grade III, G23 astrocytoma grade IV, G5 glioma grade II, G28 glioma grade III, G31 gliosarcoma, G7-G35 glioblastoma grade IV). β-actin was used as loading control. **C.** Representative Western blot showing phospho and total STAT3 protein levels in 7 human primary glioma cell lines established from the indicated cancer patients (G17, G18, G22, G24, G25, G26, G27). β-actin was used as loading control. **D.** Representative Western blot showing CB1, p-STAT3, total STAT3 protein levels in central tumor tissue **C** and in the corresponding intermediate peritumoral **(I)** and peripheral normal tissue specimen **(P)**, approximately 1cm distant from tumor mass, of one representative patient. β-actin was used as loading control. **E.** Western blot analysis for p-STAT3, total STAT3, β-catenin, p-GSK3β and MICA on whole cell extracts from indicated primary cell lines treated with control medium or SR141716 (20 μM) for 24 h. β-actin was used as control of protein loading. Panel shows a representative Western blot of 2 different experiments performed with similar results. **F.** Western Blot analysis of STAT3 phosphorylation in siCTR- and siCB1 transfected U251 and GBM17 cells (*left*, 24 h); panel on the right shows the analysis of CB1 protein levels after transfection. β-actin was used as loading control. Panel shows a representative Western blot of 3 different experiments performed with similar results.

Finally we investigated the cell signaling pathway that might mediate the response to SR141716. Since active STAT3 in tumors influences tumor survival, angiogenesis, metastasis, chemoresistance and immune evasion, too, we finally looked for a regulation of STAT3 phosphorylation targeted by CB1 inhibition. Indeed, either for Glycogen Synthase Kinase-3 (GSK-3β)-catenin pathway either, more recently, for the transcription factor STAT3 a role in the regulation of MICA/B expression has been described. Thus we investigated the possible effects of SR141716 on modulating GSK-3β and STAT3 activity in our experimental model starting from U251 glioma cell lines, while confirming it in high- and low-CB1-expressing primary cells. First western blot analysis showed that the treatment with SR141716 can increase the expression of MICA, by selectively reducing Tyr705 phosphorylation of STAT3 in U251 cells (Fig. 6A), with no detectable effect on signal pathway activated by GSK-3β activity, evaluated by measuring the phosphorylation of GSK3β and β-catenin expression. As shown in Fig. 6A and in accordance to the biological effects observed, all these molecular events were already evident after exposure to SR141716 for 18 h and more pronounced after 24 h. These preliminary results therefore indicate that SR141716 might really have dual combined activity on tumor growth and immunogenicity by a mechanism that can involve STAT3. Interestingly, this previously unrecognized finding in cancer cells, highlighted a new potential oncogenic network that led us to investigate STAT3 activity status in our tumor samples correlating them to CB1 expression levels. Interestingly, a strict and unexplored directionality CB1→STAT3 was found both in almost all tumor tissues (18/23) (Figs. 3D and 6B) and in patients primary cell lines where the levels of active STAT3 expression followed those of CB1 (Figs. 4E and 6C). Surprisingly a gradually higher CB1 expression was found from peripheral to central tissue of the dissected tumor from one representative patient and these levels inversely correlate with STAT3 activity status (Fig. 6D). To corroborate these findings we then conducted western blot analysis after 24 h treatment with SR141716 on 2 primary glioma cell lines characterized by the highest CB1 expression (GBM17, GBM18) and on 2 low- CB1-expressing cell lines (GBM25, GBM27). Specifically, we paid our attention on previously highlighted CB1-STAT3 network and its possible modulation by SR141716. As shown in Fig. 6E the treatment with SR141716 selectively reduced Tyr705 phosphorylation of STAT3 of high-CB1-expressing cells (Fig. 6E, *left*) with no detectable effect on STAT3 activation status of low-CB1-expressing cells (Fig. 6E, *right*). As control, GSK-3β-catenin pathway was marginally affected only in one patient (GBM18), that might exclude a correlation between this last pathway and SR141716 responsiveness. According to the findings on U251 glioma cell line (Fig. 6A), SR141716 increases the expression of MICA on the responsive high-CB1-expressing cells, but not on low-CB1-expressing primary cell lines. Finally, since overexpression of CB1 correlated with STAT3 activity in almost all brain tumor tissues and GBM primary cell lines analyzed, we determined if inactivation of CB1 by siRNA knockdown reversed this finding. U251 and GBM17 cell lines transfected with CB1 siRNA showed considerably less active STAT3 compared to cells transfected with scrambled siRNA (Fig. 6F). Overall these data point out to CB1-STAT3 as a new oncogenic molecular activation signature that might differentiate, alone or together with other possible regulatory elements, the response to SR141716 of high-CB1 from low-CB1 expressing cells.

## DISCUSSION

Our findings provide new insight into the physiopathology of the endocannabinoid system and in particular of CB1 receptor signaling, strengthening the new concept of CB1 as positive modulator of cell proliferation and immune escape in glioma. Indeed, proliferation-promoting functions of CB1 receptors have been already reported in neurogenesis, that is impaired in mice lacking CB1 and in wild-type mice administered with CB1 antagonist, implying that an endogenous signaling through this receptor promotes basal levels of neurogenesis *in vivo* [27]. In line with this, an antiproliferative action of CB1 antagonism in thyroid, mantle cell lymphoma, colon and breast cancers [15–20], as well as in adipocyte and in hepatic myofibroblast has been demonstrated [28–31]. Of note, our group also provided evidence that CB1 receptor inactivation inhibits angiogenesis that contributes to the growth and survival of the most aggressive and vascolarized cancers [19].

In this study, we present previously unrecognized functions of SR141716 in multimodal anti-glioma action. Here we report the upregulation of CB1 in human glioma tissues and primary cell lines where a surprising ability of SR141716 to interfere with common oncogenic processes related to cell cycle progression, apoptosis and immune escape is highlighted. Indeed, in addition to the direct cytotoxic and antiproliferative effect in established U251 glioma cell line and primary tumor cells, SR141716 also improves the immunorecognition of cancer cells by inducing the NKG2D ligand MICA/B. The documented efficacy in growth inhibition in glioma xenograft corroborated the chemotherapeutic potential of this CB1 antagonist bringing an interesting new dimension to the use of this class of drugs as effective anticancer agents. The *in vivo* major drawback in glioma therapy is, in fact, the lack of complete eradication, leading to the selection of resistant cancer cells and, as a consequence, combinatory treatment targeting glioma cells in different ways probably represents a more fruitful approach. In this context, the documented cytotoxic potential and high activity to boost competent immune surveillance mechanisms of SR141716 may represent a hopeful alternative to other established chemotherapeutics. The uncovering of STAT3 as a target of SR141716 activity, which we believe is novel in cancer, constitutes the molecular bases of this dual activity enclosed in SR141716 molecule and highlight CB1-STAT3 as a potential new oncogenic axis in the complex glioma biology.

Indeed, SR141716 impacts U251 and primary glioma cells growth and MICA/B expression through the specific inhibition of activated STAT3, a transcription factor harboring both oncogenic and immunosuppressive functions [32–34]. Of note the treatment of U251 glioma cell line with SR141716 is able to reduce the expression levels of the cell cycle regulator Cyclin D1 (Fig. 1D), of the anti-apoptotic Bcl-2, Bcl-XL (Supplementary Fig. 1) and the TGF-β1 production (Fig. 6A), all canonical targets of a deregulated STAT3 transcriptional activity in the process of carcinogenesis and tumor survival [34]. At the light of these findings the inhibition of STAT3 activation might really account not only for removal of STAT3-mediated repression of MICA [33], but also for drug effects on glioma growth and apoptosis, even though we cannot rule out an antiproliferative effect of SR141716 probably via TGF-β signaling blockage. Indeed preliminary results showed that SR141716 inhibited also phosphorylation of SMAD2/3 (Supplementary Fig. 2), central mediators of signals from TGF-β receptors. However because TGF-β signaling pathways can be Smad dependent or independent, these new findings, even though intriguing and exciting, need to be extensively underpinned in the next future.

The recent evidence that deletion of mouse astroglial CB1 receptors impairs leptin-mediated STAT3 activation [35], along with that electroacupuncture (EA) pretreatment enhances active form of STAT3 via CB1 receptor to protect against cerebral ischemia [36], support our findings corroborating the CB1-STAT3 axis as a new potential oncogenic network in brain that deserves much attention in the near future. Up to now our data highlighted, indeed, that a strict directional correlation between CB1 and STAT3 was found both in almost all tumor tissues (18/23) and in all patients primary cell lines where the levels of phosphorilated-STAT3 followed the trend of the CB1 expression (Figs. 3D and 6B, Figs. 4E and 6C), suggesting that Tyr^705^ phosphorylation of STAT3 in these cells may be dependent on the signaling through this G-protein coupled receptor (GPCR), in line with CB1 receptor knockdown experiments too (Fig. 6F). In support of this new perspective, several examples exist of GPCRs with transforming abilities, when overexpressed, in NIH 3T3 fibroblast model, for example, the MAS oncogene [37] and serotonin 1c receptors [38]. CB2 has also been described as a new transforming GPCRs in several human myeloid cell lines and primary acute myeloid leukemia (AML) samples where it has been found aberrantly upregulated [39]. Accordingly since SR141716 acts as a CB1 antagonist with properties of inverse agonism only when its target is tonically activated, we hypothesize that in pathological setting, as brain cancer, the deregulated activity of this GPCR may turn on oncogenic signals that interfere with normal proliferative response and immunesurveillance mechanisms. Indeed glioma patient samples reported high levels of CB1 expression, compared with NHA. Of interest, the extent of CB1 but not CB2 receptor protein expression was related with tumor malignancy. Thus a glioma grade II patient (relapse-free survival 38 months) and an astrocytoma grade II (relapse free survival) had lower levels of CB1, whereas most of glioblastoma IV patients, with recurrence of the tumor within 9 months from surgery, had high levels of CB1 (Supplementary Table 1). The tumor established primary cell lines confirmed the high levels of CB1 expression compared with normal human astrocytes (NHA), reinforcing the pertinence of the model used for testing SR141716 effects. However, endogenous systems that become dysregulated in cancer can be useful pharmacological targets and predictor biomarkers of the benefit from specific therapies as for example the biochemical evaluation of ER or of ERBB2, which predict the benefit from endocrine therapies and trastuzumab, respectively, in breast cancer. In our experimental model the careful selection of individual primary cancer cells with different CB1 receptor expression enables us to verify the CB1 dependency of SR141716 effects. Interestingly, we found that the degree of CB1 expression conferred a different sensitivity to SR141716 treatment, being CB1 strongly positive cells more likely to respond to SR141716 treatment *in vitro* than cells derived from low to moderate receptor levels tumors. Although this finding can be suggestive of a CB1 dependency of SR141716 activity, we cannot rule out its off-target effects, too. In our experimental model SR141716 showed its efficacy at a concentration of 20 μM that is reported to be non selective for CB1 receptor only. At this dose, SR141716 can produce also modulation and internalization of GPR55 [40], an orphan G-protein linked receptor that appears up-regulated in aggressive manner in some cancer-derived cell lines and play a pivotal role in the control of cancer cell fate [41–42]. Very recently, Moreno *et al*. described that GPR55 and CB2 can form heteromers in cancer cells and that both receptors co-participate via direct receptor-receptor interaction in the control of tumors growth [43]. Receptor heteromers involving the sister CB1 receptor have been the focus of intense research. To date CB1 has been shown to interact with other GPCRs, including dopamine D2 receptors and adenosine A2 receptors simultaneously [44], opioid receptors [45], orexin OX1 receptors [46], and angiotensin AT1 receptors [47]. Intriguingly a recent paper by Kargl et al., reported that in HEK293 cell model CB1 receptor can physically interact with GPR55 and modulate each other signaling properties [48]. Specifically GPR55 signaling is inhibited in the presence of SR141716 inactivating-CB1 receptors, highlighting an important cross-antagonism action for this molecule. On the other hand, the findings that the high selective CB2 antagonist SR144528 did not affect glioma cells proliferation in the same experimental conditions used for CB1 antagonist SR141716 (*data not shown*), excluded its non specific effects on CB2 receptors. Accordingly to the new strict and previously unexplored directionality CB1→STAT3, in our experimental model the differential effect of SR141716 on primary glioma growth occurred in concert with the differential changes of CB1 expression in tumor tissues and consequently with basal activated STAT3 levels. The only apparent paradox of the same degree of susceptibility of high- and low-CB1-expressing tumor cells to NK cell-mediated killing (Fig. 5) could be explained through the possible induction of other NK activating factors by SR141716 treatment. In this respect apoptosis proteome profiler array analysis (Supplementary Fig. 1) showed that SR141716 killing of U251MG *in vitro*, at least in part, is dependent on induction of tumor necrosis factor (TNF)-related apoptosis-inducing ligand and its ligand death receptor 4 (DR4). NK cells are known to express functional TNF-related apoptosis-inducing ligand [49] and it is indeed possible that a part of the enhanced NK lysis we observed after SR141716 is caused by induction of DR4 or other member of this family on the target cells for the control of low grade malignancy. Additionally, STAT3 has been shown to act also as a transcriptional repressor of p53 [50] that, apart from its cell-autonomous effects, activates the innate immune response against cancer cells stimulating the expression of ULPB2 [51], in human tumor cells of different origin and so, the lower level of active STAT3 found in low-CB1-expressing tumor cells may be responsible of a major amount of this immunogenic ligand on their cell surface. Overall regarding the potential patient stratification, CB1 detection alone or combined with other conventional biomarkers may allow to select which particular individuals are potentially responsive to drug administration. Taking all this in account, our findings showing a direct and NK-mediated antitumor effect exerted by SR141716 in glioma cells, its proved efficacy *in vitro* and *in vivo* (Fig. 3A), the weak and poor effects on healthy brain cells (Figs. 1–2) and the well know pharmacological ability to penetrate throughout the CNS and blood-brain barrier are adjunct values that may led to a renewed interest toward SR141716 as a chemotherapeutic agent capable of inducing multimodal glioma destruction. Because different aspects of glioma biology have been separately targeted with very limited success, we speculate that to obtain significant clinical results a great therapeutic effort should be aimed to found new compounds which enclose in the same molecule cytotoxic potential and high activity to boost competent immune surveillance mechanisms. As here showed, CB1 antagonism capable of tumoral cell division' control while making the glioma immunovisible and engaging the immune system to fight it may represent a hopeful alternative to other established chemotherapeutics approaches.

## MATERIALS AND METHODS

### Reagents and Abs

SR141716 (Rimonabant) was kindly donated by Sanofi-Aventis. It was dissolved in DMSO and added to cells cultures at the indicated concentrations. The following mAbs were used for immunostaining or as blocking Abs: anti-MICA/B/PE, anti-CD56/PerCP/Cy5.5, anti-CD69/PE, LEAF™ purified anti-human MICA/MICB (6D4), LEAF™ purified Mouse IgG2a κ Isotype Ctrl, anti-AnnexinV/FITC, anti-CD68/FITC, anti-MDR/Cyc, anti-Ki67/PE were purchased from BioLegend (San Diego, CA, USA), anti-A2B5/PE, CD15/APC, Ep-CAM/APC, CD24/PE, CD133/PE from Miltenyi Biotec (Calderara di Reno, Italy); MHC-I/FITC from SantaCruz (SantaCruz, CA, USA); anti-CD3/FITC, anti-HLA, B, C/APC, anti-CD45/Cyc from BD Pharmingen (San Jose, CA). Anti-ULBP1/PE, Anti-ULBP2/ULBP6/APC, anti-ULBP3/PE, anti-RAET1E/ULBP4/PerCP, anti-MICA/PE, anti-MICB/APC, goat IgG/PerCP, IgG1/PE, or mouse IgG2A/APC isotype control were purchased from R&D Systems (Minneapolis, MN).

z-VAD-fmk was obtained from R&D Systems (Minneapolis, MN, USA).

In fluorescence microscopy rabbit polyclonal anti-human MICA (Abcam, Cambridge, UK) and Fluor^®^ 488 donkey anti-rabbit IgG secondary antibody (Molecular Probes^®^-Invitrogen, Paisley, UK) were used.

For western blot analysis the following antibodies were used: rabbit monoclonal anti-human beta catenin, rabbit polyclonal anti-human cannabinoid receptor I, rabbit polyclonal anti-human cannabinoid Receptor II, rabbit polyclonal anti-human MICA, rabbit polyclonal to SMAD 1/5/8/9 and rabbit polyclonal anti-human β-actin were purchased from Abcam (Cambridge, UK), mouse monoclonal anti-human Cyclin D1 and mouse monoclonal anti-human p27 (Kip1) were purchased from BD Pharmingen (San Jose, CA), mouse monoclonal anti-human α-tubulin from Sigma-Aldrich Inc. (St Luis, MO, USA), rabbit monoclonal anti-human phospho- GSK-3β (p-GSK-3β; Ser9), rabbit monoclonal anti-human Phospho-Smad2 (Ser465/467)/Smad3 (Ser423/425), rabbit polyclonal Smad2/3, rabbit polyclonal antibodies to phosphorylated Smad1/5/8, rabbit polyclonal anti-human cleaved caspase-3, rabbit polyclonal anti-human caspase-3, rabbit polyclonal anti-human phospho-STAT3 (p-STAT3; Tyr705) and rabbit monoclonal anti-human STAT3 were purchased from Cell Signaling Technology (Danvers, MA). Secondary HRP-linked goat anti-mouse or goat anti-rabbit IgG, were also purchased from Cell Signaling Technology (Danvers, MA).

### Cells and clinical samples

Normal Human Astrocytes (NHA) are normal human cells derived from human brain tissue and were cultured in recommended medium AGM™ BulletKit™ (Lonza). The human glioma cell lines U343MG (U343), U87MG (U87), U251MG (U251) and T98G (T98) were obtained from CLS Cell Lines Service GmbH (Eppelheim, Germany) or were kindly provided by Dr. Daniela Parolaro (University of Insubria, Italy).

Small pieces of brain tissue containing tumor were collected at the time of craniotomy for tumor resection at the Neurosurgery Service of “G. Rummo” Medical Hospital (Benevento, Italy) and divided into a portion immediately processed to generate primary tumor cell lines and a portion stored at −80°C for subsequent molecular characterization (RNA, DNA and protein extraction). A second sample from each patient was also taken for clinical diagnosis performed by expert neuropathologists in accordance with the International Classification of CNS tumors drafted under the auspices of the World Health Organization (WHO). The tumors were diagnosed as astrocytoma (WHO grade I-III; *n* = 3), glioma (WHO grade II, *n* = 2) or glioblastoma multiforme (WHO grade IV; *n* = 18). There were not significant differences by gender, or age between the different groups (Supplementary Table 1). All tissue samples were collected in accordance with the ethical standards of the Institutional Committee. The patients had been informed about the establishment of cellular models from their tumour and had given informed consent in written form. The preparation of adherent primary cultures of brain tumor cells (designated as GBM*n*) was conducted through gentleMACS dissociation of brain tumor samples using BTDK Brain Tumor Dissociation Kit (Miltenyi Biotec, Calderara di Reno, Italy) and kept in culture in DMEM F12 supplemented with 15% heat-inactivated fetal bovine serum (Euroclone), 2% L-glutamine, 1% antibiotic mixture, 1% sodium pyruvate, 1% non-essential aminoacids (Euroclone). Experiments were performed using passages 2–6 of these cells.

Human glioma cell lines were cultured in EMEM (Lonza) supplemented with 10% heat-inactivated fetal bovine serum (Euroclone), 1% L-glutamine, 1% antibiotic mixture, 1% sodium pyruvate, 1% non-essential aminoacids (Euroclone).

Human primary Natural killer (NK) cells were negatively selected from peripheral blood mononuclear cells (PBMC) of healthy donors by immunomagnetic procedure (NK-cell isolation kit; Miltenyi Biotec, Calderara di Reno, Italy) and cultured for different time intervals in RPMI 1640 (Invitrogen, San Diego, CA, USA) supplemented with 2 mM L-glutamine, 50 ng/ml streptomycin, 50 units/ml penicillin, and 10% heat-inactivated fetal bovine serum (Hyclone Laboratories, Logan, UT, USA) in the presence of IL-2 at 100 U/ml alone (Roche Applied Science, South San Francisco, CA, USA). All donors gave written informed consent in accordance with the Declaration of Helsinki to the use of their residual buffy coats for research purposes, with approval from the University Hospital of Salerno Review Board. Peripheral blood mononuclear cells (PBMCs) were isolated over Ficoll-Hypaque gradients (lymphocyte separation medium; MP Biomedicals, Aurora, OH, USA).

All cell cultures were maintained at 37°C in humidified 5% CO2 atmosphere.

### mAbs and cytofluorimetric analysis

Immunofluorescence and flow cytometry analysis of a panel of markers (A2B5, CD24, CD15, Ep-CAM, MHC-I, CD133 from Miltenyi Biotec, Calderara di Reno, Italy; MDR, Ki67, CD68 from eBiolegend; CD45 and HLA-B-C from BD Pharmingen) was done in all the cell lines used in this study to corroborate that they had the features of human glioma cells. NK purity and phenotype were assessed through the staining with fluorochrome-conjugated monoclonal antibodies (mAbs) against CD56, CD3, CD69, as well as the corresponding isotype immunoglobulin of control (BioLegend, San Diego, CA, USA). For the evaluation of MICA/B expression, U251 cells and GBM primary glioma cell lines were plated into p60 tissue culture plates at a densities of 20 × 10^3^ cells/cm^2^ and were allowed to grow for 24 h. Afterwards cells were washed with PBS and treated with vehicle or test substance in complete medium. Following a 24 h incubation period, immunofluorescence staining was performed. Briefly 2 × 10^5^ tumor cells were stained with the indicated MICA-, MICB-, MICA/B-, MHC class I or ULBP1–4-specific mAbs or the respective isotype controls followed by flow cytometric analysis. Cells were fixed in 1% formaldehyde for data analysis. Sample fluorescence was measured by the Fluorescence-Activated Cell Sorter FACScalibur apparatus (Becton Dickinson, USA) and data were analyzed by using the Cell- Quest Pro software (Becton Dickinson, San Jose, CA). Data are expressed as logarithmic values of fluorescence intensity.

### Immunofluorescence staining

GBM primary cell lines were plated on slides in 12 well plates at a densities of 10 × 10^3^ cells/cm^2^ and were allowed to grow for 24 h. Afterwards cells were washed with PBS and treated with vehicle or SR141716 in complete medium. Following a 24 h incubation period, cells were fixed with 4% parafolmadehyde (PFA) for 15 minutes, washed and blocked with 4% bovine serum albumin (BSA) for 1 h at room temperature. Cells were then incubated with anti-MICA primary antibody (Abcam, Cambridge, UK) at final concentration of 20 μg/ml at 4°C overnight. Immunofluorescence staining was obtained by incubating for 90 minutes with Alexa Fluor^®^ 488 donkey anti-rabbit IgG secondary antibody (A21206, Molecular Probes^®^) at final concentration of 2 μg/ml. The nuclei were counterstained with DAPI (1:2000) (Molecular Probes^®^-Invitrogen, Paisley, UK) and slides were mounted using Mowiol Coverslip Mounting solution for Fluorescence Microscopy (Fluka, – through Sigma-Aldrich Inc., St Luis, MO, USA). Cells were examined under fluorescent microscope and then analyzed through cellSens Imaging Software (Olympus, Tokyo, Japan).

### Determination of glioma cell proliferation

Glioma cells or normal human astrocytes (NHA) (4 × 10^3^/well), were cultured for 24 h into 96-well plates before addition of SR141716 at the indicated concentrations and cultured for additional 72 h at 37°C. Cell proliferation was evaluated by measuring BrdU incorporation into DNA (BrdU colorimetric assay kit; Roche Applied Science, South San Francisco, CA, USA). Newly synthesized BrdU-DNA was determined on an ELISA plate reader (ThermoScientific) at 450 nm. All experiments were performed in triplicate, and the relative cell growth was expressed as a percentage comparison with the untreated control cells.

### Cell cycle analysis

U251 glioma cell lines were plated in 100-mm dishes. To synchronize cells at the G1/S interface, they were serum starved in medium with 0.5% serum for 18 h. Cells were treated with SR141716 (10 and 20 μM). After 48 h, the cells were collected, fixed in 70% ethanol, and kept at −20°C overnight. Propidium iodide (PI; 50 μg/ml) in PBS containing 100 U/ml DNase-free RNase was added to the cells for 15 min at room temperature. The cells were acquired by a FACSCalibur flow cytometer (BD Biosciences, San Jose, CA, USA). The analysis was performed with ModFit LT v3.2 (Verity Software House, Inc., Topsham, ME, USA); 10, 000 events, corrected for debris and aggregate populations, were collected.

### Apoptosis analysis

Quantitative assessment of apoptosis of U251 glioma cell lines was analyzed by anti-human Annexin V (BioLegend, San Diego, CA, USA) and PI staining. Briefly, cells grown in 100-mm dishes for 72 h in EMEM containing 2% FBS were harvested with trypsin and washed in PBS. The cells were resuspended in Annexin V binding buffer (10 mM HEPES/NaOH, pH 7; 140 mM NaCl; and 2.5 mM CaCl_2_) and stained with Annexin V-FITC for 20 min at room temperature (RT) and then with PI at RT for additional 15 min in the dark. The cells were acquired by flow cytometer within 1 h after staining. At least 10, 000 events were collected, and the data were analyzed by Cell- Quest Pro software (Becton Dickinson, San Jose, CA). Data are expressed as logarithmic values of fluorescence intensity.

### Real-time PCR

Total RNA was isolated from 5 × 10^6^ cells using TRIzol^®^ reagent (Invitrogen, Paisley, UK), according to the manufacturer's instructions. Complementary DNA (cDNA) was transcribed using SuperScript II Reverse Transcriptase (Invitrogen, Paisley, UK), starting from 1 μg/μl of high pure RNA and samples were tested in triplicate using the SsoFast EvaGreen reagents (Bio-Rad).

Primer sets were the following:

MICAFw–GACTTGACAGGGAACGGAAA, MICARev–CAGGTTTTGGGAGAGGAAGA; MICBFw–CAGCTACTGGGTCCACTGGT, MICBRev–GTTGGTCATGATCCCTTTGC; CB1Fw–CTTCACGGTCCTGGAGAACC, CB1Rev–ACCCCACCCAGTTTGAACAG and the house keeping gene was the β2-microglobulin: β2Fw-CCTGGATTGCTATGTGTCTGG, β2Rev–GGAGCAACCTGCTCAGATACA or β-actin: ActinFw–CACTGTGCCCATCTACGAGG, ActinRev–TGGCCATCTCTTGCTCGAAG; ULBP1Fw-CGGTGCTAATGGATGGAACT, ULBP1Rev-TGGTCAGTGCATCAAAAGGA; ULBP2Fw-TCAAACTCGCCCTTCTGTCT, ULPB2Rev-GTGCAGGAATTCCATCAGGT; ULBP3Fw–CTGGGGAAAACAACTGGAAA, ULBP3Rev-ATCGAAGCTGAACTGCCAAG; ULBP4Fw–CCCTGACTTCTAGCCCTGTG, ULBP4Rev-AGAAGACCTGCGCTTCACAC; GAPDHFw–CGCTCTCTGCTCCTCCTGTTC, GAPDHRev-TTGACTCCGACCTTCACCTTCC.

qRT-PCR protocol was: pre-heating step for 3 minutes at 95°C, then 40 cycles at 95°C for 10 seconds and 60° for 30 seconds and last end-step at 65°C for 10 seconds. Finally, results were analyzed with 2^−ßßCt^ method.

### Flow cytometric assay of NK-cell cytotoxicity

Cytotoxicity assays were performed using the fluorescent carboxy-Fluorescein Diacetate c'FDA NK assay in which cell lysis was analyzed by flow cytometry using the protocol described elsewhere [52]. Briefly, U251 or primary glioma target cells, stimulated as indicated in the text, were labeled by incubation at 1 × 10^6^/ml in PBS with 3 μM c'FDA (Molecular Probes-Invitrogen, Paisley, UK). IL-2 activated effector NK cells were mixed with and titrated on the target cells and incubated at 37°C in a humidified 5% CO2 incubator for 4 h. In blocking experiments, LEAF™ purified anti-human MICA/MICB (6D4) and LEAF™ purified Mouse IgG2a κ as isotype control were added at 10 μg/ml 2 h before starting co-incubation effector:target and maintained in culture for additional 4 h till to the end of the experiment. All determination were made in triplicate, and E:T ratios ranged from 6:1 to 1:1, as indicated.

### Determination of IFN-γ and TGF-beta1 levels by ELISA

For IFN-γ secretion measurement, human purified NK cells were stimulated with IL-2 (100 IU/ml). After 72 h, activated NK cells were washed twice and mixed with untreated or SR141716-treated glioma target cells in 200 μl of complete medium. Cells were incubated for the indicated time. Thereafter, supernatants were collected and stored at −20°C pending measurement. The concentration of IFN-γ was measured by ELISA assay according to manufacturer's specification (R&D Systems, Minneapolis, MN). For TGF-beta1 secretion measurement, human primary glioma cell lines were plated into p60 tissue culture plates at a densities of 20 × 10^3^ cells/cm^2^ and were allowed to grow for 24 h. Afterwards cells were washed with PBS and treated with vehicle or test substance in complete medium. After 24 h supernatants were collected and stored at −20°C pending measurement. The concentration of TGF-beta1 was measured by ELISA assay (LEGEND MAX™ Total TGF-β1 ELISA Kit, BioLegend, San Diego, CA, USA). Cells were treated in triplicates and ELISA were done in duplicates for each cell sample.

### Western blot (WB) analysis

For analysis of protein levels from cells they were grown in p60 tissue culture plates at a density of 2 × 10^4^ cells/cm^2^ for 24 h. Tumor cells were then incubated with vehicle or SR141716 as indicated in the text. After incubation cells were washed with PBS, harvested and lysed in ice-cold RIPA lysis buffer (50 mM Tris-HCl, 150 mM NaCl, 0.5% Triton X-100, 0.5% deoxycholic acid, 10 mg/ml leupeptin, 2 mM phenylmethylsulfonyl fluoride, and 10 mg/ml aprotinin). Tumor pieces were disrupted for protein extraction by gentle homogenization (Potter-Elvehjem Pestle) in cold RIPA buffer. After removal of cell debris by centrifugation (14, 500 g for 20 min at 4°C), the proteins were estimated and then analyzed using the protocol described elsewhere [53].

### Mice experiments

We used U87MG cell line in the tumor xenograft models because this cell line has been widely used due to their tumorigenicity in nude mice. The U251MG cells did not show any tumorigenicity in nude mouse in the initial attempts to establish xenograft model.

Athymic CD1-deficient nude mice were purchased from Charles River Laboratories (Sulzfeld, Germany). Female mice of 6–8 weeks old were used in all the experiments. The experiments were performed according to NIH guidelines, Guide for the care and Use of laboratory Animals. Mice were bred at Animal research Facility of Biogem scarl, Institute for Genetic Research “Gaetano Salvatore”, (Ariano Irpino Avellino, Italy) in accordance with Institutional Statement for the Use of Animals in cancer research.

U87 luciferase expressing glioblastoma cells (luc-U87) were furnished by Biogem scarl. Before the implant the cells were expanded, evaluated for contaminations and vitality, counted and resuspended in M199 medium. Cells were inoculated subcutaneously (s.c) on the right flank region at a concentration of 5 × 10^6^/100 μl/mouse. Treatments began 9 days after injection. The day before the first administration (day -1) mice were subdivided in tumor volume (TV) homogeneous groups of treatment, (*N* = 10–12 each) in the following experimental groups: control (peritumoral injection of vehicle, 2 times a week for 4 weeks) and SR141716 (peritumoral administration of 1mg/Kg SR141716, 2 times a week for 4 weeks). Mice were daily monitored for clinical signs and mortality. Body weight recordings were carried out biweekly. Tumor growth was monitored biweekly with a Mitutoyo caliper. The formula TV (mm^3^) = [length (mm) x width (mm)^2^]/2 were used, where the width and the length are the shortest and the longest diameters of each tumor, respectively. The end of the experiment was fixed at 6weeks after the injection. After 35 and 41 days from treatment beginning, 4 animals for each group were sacrificed by cervical dislocation. Explanted masses were weighted, photo recorded and fixed in formalin 10% for immunohistological analysis.

### Transfection

U251MG cells and GBM17 primary cell line (75% confluent) were transfected with control siRNA (100 nM) or CB1 siRNA duplexes (100nM) using Lipofectamine 2000 Transfection reagent (Invitrogen, Paisley, UK) according to the manufacturer's protocol. A 21-nucleotide small interfering RNA duplex (Dharmacon Research) was used for specific silencing of CB1 (siRNA-CB1), covering the sequence sense 5′-CCCAAGUGACGAAAACAUU-dTdT-3′.

### Immunohistochemistry

Criosections (0, 7 mm) of luc-U87 explanted masses prepared at day 35 and 41 were fixed in acetone and blocked with 2% normal rabbit serum and 2% BSA. Tissue sections were stained with antibodies (1:50) to MICA (Becton Dickinson, San Jose, CA). A biotinylated anti-rat secondary antibody (Zymed, San Francisco, CA) was used at 1:150. Avidin-biotin complex was added, and the staining was developed with diaminobenzidine.

For the evaluation of MICA staining, a score combining the staining intensity and distribution was used. Staining intensity and the distribution for MICA was scored as 0 (no staining), 1 (predominantly unstained with smaller stained areas), 2 (stained and unstained areas are about equally large), 3 (predominantly stained tissue with smaller unstained areas), 4 (all xenografted cells are moderately stained), and 5 (all xenografted cells are strongly stained). The MICA staining score are the mean values of five to eight scored samples of tumor tissues belonging to each group [54]. Slides were scored without any pre-clinical information, and the final immunostaining score reported was the average of two independent pathologists, both with experience in the IHC analysis.

### Statistical analysis

Statistical analysis was performed in all the experiments shown by using the GraphPad prism 6.0software for Windows (GraphPad software). For each type of assay or phenotypic analysis, data obtained from multiple experiments are calculated as mean ± SD and analyzed for statistical significance using the 2- tailed Student *t*-test, for independent groups, or ANOVA followed by Bonferroni correction for multiple comparisons. *P* values less than 0.05 were considered significant. *P < 0.05, **P < 0.01 and ***P < 0.001.

## SUPPLEMENTARY FIGURES AND TABLE


