# Supplementary material for: Cannabinoid receptor CB1 regulates STAT3 activity and its expression dictates the responsiveness to SR141716 treatment in human glioma patients' cells
Source: Oncotarget. 2015 May 11;6(17):15464–81. doi: 10.18632/oncotarget.3895 (PMC4558164; doi:10.18632/oncotarget.3895)
Supplement: Supplementary file 1 [file oncotarget-06-15464-s001.pdf]

# SUPPLEMENTARY FIGURES AND TABLE

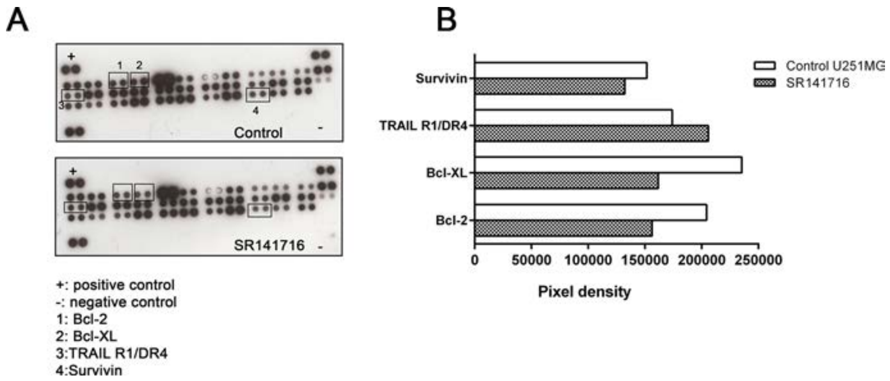


**Supplementary Figure S1: Effect of SR141716 treatment on the apoptotic protein expression profiles of U251 glioma cell line.** U251 cells were treated with SR141716 20 μM for 24 h and cell lysates were used to determine different protein levels through a human array kit/proteome profiler. **A.** Panel shows a representative proteomic membrane analysis with indication of the modified proteins. **B.** Densitometric analysis of the membrane spots reported as pixel densities.

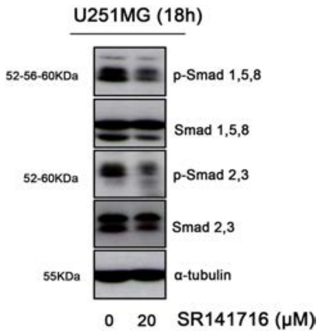


**Supplementary Figure S2: Effect of SR141716 treatment on Smad-dependent TGF-β signaling pathway in U251 glioma cell line.** U251 cells were treated with SR141716 20 μM for 18 h and cell lysates were subjected to western blot with anti-phospho-SMAD2/3, anti-phospho-SMAD1/5/8 and the corresponding anti-total-SMAD2/3 and anti-total-SMAD1/5/8 antibodies. α-tubulin monoclonal antibody was used as an internal loading control.

**Supplementary Table S1.** Clinical characteristics of the patients' cohort

| Patient | Survival (months) | Recurrence (months) | Clinical Stage | Age | Gender | Treatment post-lesion |
|---|---|---|---|---|---|---|
| **GBM2** | 15 | 9 | Astro III | 44 | M | Chemotherapy-Radiotherapy |
| **GBM5** | 17 | 38 | Glioma II | 69 | F | Chemotherapy |
| **GBM7** | 16 | free | IV | 49 | M | Chemotherapy-Radiotherapy |
| **GBM15** | 9 | 9 | IV | 73 | M | STUPP Protocol |
| **GBM16** | 9 | free | Astro II | 31 | M | Wait and See |
| **GBM17** | 8 | 9 | IV | 48 | F | Chemotherapy |
| **GBM18** | 7 | 4 | IV | 74 | M | Chemotherapy |
| **GBM20** | 6 | free | IV | 64 | M | STUPP Protocol |
| **GBM21** | 10 | 4 | IV | 57 | M | STUPP Protocol |
| **GBM22** | 8 | free | IV | 72 | F | STUPP Protocol |
| **GBM23** | 6 | free | Astroblastoma | 27 | M | STUPP Protocol |
| **GBM24** | 5 | residual | IV | 65 | F | STUPP Protocol |
| **GBM25** | 5 | 3 | IV | 77 | F | STUPP Protocol |
| **GBM26** | 2 | residual | IV | 66 | M | STUPP Protocol |
| **GBM27** | 2 | residual | IV | 64 | F | STUPP Protocol |
| **GBM28** | 8 | 9 | III | 52 | M | STUPP Protocol |
| **GBM29** | 8 | 5 | IV | 43 | M | Fotemustine |
| **GBM30** | 4 | 1 | IV | 58 | F | STUPP Protocol |
| **GBM31** | 5 | residual | Gliosarcoma | 66 | M | STUPP Protocol |
| **GBM32** | 7 | 7 | IV | 48 | M | Fotemustine |
| **GBM33** | 5 | free | IV | 48 | F | STUPP Protocol |
| **GBM34** | 6 | 1 | IV | 54 | M | STUPP Protocol |
| **GBM35** | 10 | free | IV | 41 | M | STUPP Protocol |
